# Investigating media that support red wolf (*Canis rufus*) sperm viability and capacitation *in vitro*

**DOI:** 10.1530/RAF-20-0042

**Published:** 2020-12-28

**Authors:** Jennifer B Nagashima, Marcia de Almeida Monteiro Melo Ferraz, Sarah H Kamen, Nucharin Songsasen

**Affiliations:** 1 Smithsonian Conservation Biology Institute, National Zoological Park, Front Royal, Virginia, USA; 2 Gene Center, Ludwig-Maximilians University, Munich, Bayern, Germany; 3 Department of Biological and Environmental Sciences, Longwood University, Farmville, Virginia, USA

**Keywords:** sperm, capacitation, red wolf, assisted reproduction

## Abstract

The red wolf is a critically endangered canid, with ~250 and ~20 individuals in the *ex situ* and reintroduced wild populations, respectively. Assisted reproductive technologies such as sperm cryopreservation and *in vitro* fertilization therefore represent critically-needed tools to manage these populations. However, the motility of post-thaw red wolf sperm rapidly declines during *in vitro* incubation, hindering the ability to develop these technologies. In this study, we evaluated the influence of several culture media (a modified canine capacitation medium (mCCM), a modified North Carolina State University-23 medium (mNCSU-23), a synthetic oviductal fluid (SOF), a fertilization Tyrode’s medium base or Fert-TALP (FERT), and a TRIS-based buffer (TRIS)) on the survival and capacitation of red wolf sperm during extended (18 h) incubation at 38.5°C and 5% CO_2_. Red wolf sperm motility averaged (±s.e.m.) 73.8 ± 7.1% at the time of collection, and was better maintained over 4 h incubation in mCCM (55.0 ± 9.8%) and mNCSU-23 (54.7 ± 10.4), compared to mSOF (43.8 ± 8.3%), FERT (30 ± 10.5), and TRIS (16.4 ± 4.1%) solutions. Patterns of tyrosine phosphorylation signal, as assessed via immunocytochemistry, indicated induction of capacitation between 2 and 4 h *in vitro* culture. Tyrosine phosphorylation signal was particularly robust in mCCM and mNCSU-23 incubated sperm, although significant acrosome exocytosis was not observed in response to progesterone supplementation after 3 h incubation in any of the media. In sum, results indicate mCCM and mNCSU-23 are promising base media for the *in vitro* incubation and capacitation of red wolf sperm, for assisted reproduction applications.

**Lay summary:**

Development of assisted reproductive technologies such as *in vitro* fertilization and artificial insemination is of high importance to the genetic management of critically endangered species such as the red wolf (*Canis rufus*). However, these technologies require the ability to maintain sperm viability and function during extended incubation, which has not been successful for the red wolf thus far. In this study, various culture media developed for sperm/egg/embryo culture in large mammalian species were evaluated for their ability to maintain red wolf sperm motility under physiological incubation conditions. Media and conditions previously utilized for domestic dog sperm were found to best support sperm incubation and capacitation (process of becoming competent to fertilize an egg) in the red wolf, representing a key step for future development of assisted reproductive technologies for the species.

## Introduction

The red wolf is a critically endangered American canid, with fewer than 20 individuals in the reintroduced population and approximately 250 managed individuals in the Association of Zoos and Aquariums (AZA) Species Survival Plan population (Lasher 2020). Specifically, the most recent red wolf Population Viability Analysis determined that, in the absence of ongoing genetic management or population expansion, the AZA SSP population will retain only ~75% gene diversity in the next 125 years, and the reintroduced population is projected to go extinct over the next 8–37 years (Faust *et al.* 2016). As result, it is vital to the long-term survival of the species that every valuable animal is represented in the next generation, to maintain genetic variability. While ideally this could be accomplished via natural breeding, this comes with the risk of transporting individuals to different locations for breeding and failed reproduction due to sexual incompatibility. In recent decades, assisted reproductive technologies (ART), especially artificial insemination (AI) have played significant roles in the management of many endangered species, including the black-footed ferret (Howard *et al.* 2016), the giant panda (Huang *et al.* 2012) and whooping crane (Blanco *et al.* 2009). Though not yet regularly implemented in canid breeding management programs, there have been successful reports of artificial insemination in gray wolves (Asa *et al.* 2006), blue and red foxes (Nyberg 1980, Farstad *et al.* 1992), and critically endangered Mexican gray wolves (Mossotti 2017). As such, there is significant interest and potential for AI and other ART to support endangered canid conservation efforts (Comizzoli *et al.* 2009, Amstislavsky *et al.* 2012).

Previous assisted reproduction research in red wolves has characterized semen and optimized sperm cryopreservation and thawing protocols (Goodrowe* et al.* 1998, Koehler *et al.* 1998, Franklin *et al.* 2018, Ferraz *et al.* 2020). For example, though the acrosomal membranes of red wolf sperm are highly sensitive to cryodamage (Goodrowe *et al.* 1998), investigations on cooling strategies (Goodrowe *et al.* 2001), extender osmolarity and egg yolk concentration (Lockyear *et al.* 2009), and cryoprotectant and post-thaw culture temperatures (Franklin *et al.* 2018), have improved survival and motility post-thaw. However, sperm motility/viability has been shown to drop precipitously during post-thaw incubation in the TRIS-based extender commonly used in cryopreservation (Lockyear *et al.* 2009, Franklin *et al.* 2018). As such, improvements in sperm incubation conditions and/or cryopreservation methods are necessary for future use of banked semen. Specifically, our ability to fully evaluate the quality and longevity of post-thaw semen as well as to develop *in vitro* fertilization technologies for this species requires long-term sperm incubation capacity.

Similarly, additional studies into the fertilizing capacity of red wolf sperm, in general, are necessary. Thus far, only one study has described the binding and penetration of fresh and frozen/thawed red wolf sperm to domestic dog oocytes, as an assay of the fertilizing potential of red wolf sperm (Goodrowe *et al.* 2001). *In vivo*, ejaculated sperm are not immediately capable of fertilizing an oocyte, and first must undergo capacitation. Capacitation is the process by which a sperm becomes developmentally competent to fertilize (Chang 1951, Austin 1952) and is characterized by phosphorylation of tyrosine residues in sperm proteins, the development of hyperactive motility, and the ability of the sperm to undergo acrosomal exocytosis (Visconti *et al.* 1995). Once in the female reproductive tract, sperm are exposed to stimuli that support efflux of sterols from the plasma membrane, removal of decapacitating seminal plasma proteins, and changes in intracellular pH and calcium, which cumulatively promote capacitation (Visconti *et al.* 1995). *In vitro* capacitation can be stimulated by compounds such as BSA, which results in cholesterol efflux from the sperm membrane and increased membrane fluidity (Cross 1998). Sodium bicarbonate, which facilitates the rise of sperm cyclic adenosine 3’,5’-monophosphate (cAMP) and intracellular pH (Visconti *et al.* 1999, Demarco *et al.* 2003), supports protein tyrosine phosphorylation, lipid membrane remodeling, and acrosome exocytosis (Buffone *et al.* 2014). Sperm subsequently exposed to factors present in the oviductal environment, including progesterone or zona pellucida proteins, undergo acrosomal exocytosis *in vitro* if capacitation has been achieved (Roldan *et al.* 1994, Brewis *et al.* 2001). This acrosome reaction, in turn, is necessary for sperm penetration and fusion with the oocyte and fertilization (Wassarman 1999). Thus, *in vitro* sperm incubation and capacitation methods are key to evaluate the fertilizing capacity of red wolf semen, and for the future development of IVF in the species.

Toward these goals, a variety of ‘capacitating’ and gamete/embryo holding media have been developed for other species which can be evaluated for utility in supporting red wolf sperm *in vitro*. Domestic dog IVF utilized a modified canine capacitation medium (mCCM) (Nagashima *et al.* 2015), which had been developed specifically to promote sperm capacitation (Mahi & Yanagimachi 1978). Fert-TALP (FERT), a modified Tyrode’s balanced salt solution, is traditionally used in bovine IVF (Parrish 2014). A medium developed for porcine embryo culture, named North Carolina State University-23 (NCSU-23 (Petters & Wells 1993)), has also been utilized for IVF in the domestic dog (Nagashima *et al.* 2015). In the latter study, a modified NCSU-23 containing magnesium chloride and sodium pyruvate maintained dog sperm viability *in vitro* for over 24 h (Nagashima, personal observation). Synthetic oviductal fluid (SOF), was originally developed for ovine embryo work, but has also been utilized in domestic dog oocyte culture (Hewitt & England 1999). Finally, TRIS extender, the medium used in red wolf sperm cryopreservation, has also been utilized in the post-thaw incubation of red wolf semen (Lockyear *et al.* 2009, Franklin *et al.* 2018). Importantly, incubation of both fresh (Goodrowe *et al.* 1998) and frozen–thawed (Franklin *et al.* 2018) red wolf sperm in TALP- and TRIS-based media, respectively, at 37°C resulted in a more rapid decline of sperm motility/viability, compared with ambient temperatures. Therefore, the objective of this study is to identify a base media (mCCM, FERT, mNCSU-23, mSOF, or TRIS) supportive of red wolf sperm incubation at physiological temperatures (38.5°C), toward the goal of developing a capacitation protocol for red wolf sperm.

## Materials and methods

### Animals

Adult (ages 2–9 years old) male red wolves housed at AZA Species Survival Plan facilities in the Eastern United States were selected for collection based on health status, mean kinship value, and geographic location. Animals were exposed to natural photoperiod, fed diets consisting of commercially available dry dog food and/or whole carcass, with water provided *ad libitum*. All procedures were done in accordance with the AZA SSP and USFWS guidelines and Smithsonian Institution International Animal Care and Use Committee approval (IACUC #18-05).

### Sperm collection and processing

A single collection attempt was made per animal in the 2018 and 2019 breeding seasons (January–March), for a total of eight ejaculates collected from six individuals (aged 3–9 years, with two wolves collected in both 2018 and 2019 seasons). Wolves were fasted 24 h prior to semen collection. On the day of semen collection, animals were captured, transferred to a crate and transported a short distance from their outdoor enclosure to an indoor treatment room, wherein wolves were anesthetized by veterinary staff. Typically, red wolves were anesthetized using butorphanol (0.4 mg/kg body weight IM) and medetomidine (0.4 mg/kg IM). Prior to the start of electroejaculation, the rectum was evacuated using a well-lubricated gloved hand and the penis carefully exteriorized, washed thoroughly with sterile saline and wiped dry.

Red wolf semen was collected via electroejaculation as previously described (Franklin *et al.* 2018). Briefly, to avoid urine contamination in semen samples, the urinary bladder was emptied by inserting a 5 Fr, 55.8 cm long polypropylene catheter (Covidien/Kendall) into the urethra, the urine aspirated and the bladder flushed with warmed sterile saline. Thereafter, a lubricated rectal probe (1.9–2.1 cm) was inserted and 60–90 electrical stimulations (2–6 V) were applied over a period of 20 min. The intensity of the stimulation was adjusted as appropriate based on the individual animal’s physical response. Ejaculates were collected into sterile polypropylene specimen cups, pooled, and a small volume was assessed for sperm concentration (via hemocytometer), motility, and pH. In cases where semen volume or concentration was low, the second round of stimulations was attempted. Animals were allowed to recover from anesthesia under the care of veterinary staff.

Semen fractions with no evidence of urine contamination (based on color and pH 6–8) and presence of sperm from all stimulation rounds were pooled per individual, then centrifuged at 500 ***g*** for 3 min to pellet the sample/remove seminal plasma. A portion of the sperm was then resuspended/washed in 1 mL warmed sterile PBS (Sigma-Aldrich 59331C) prior to *in vitro* incubation and capacitation evaluations.

### Sperm incubation

Following collection and washing, sperm were incubated in each of five different base media at a final concentration of 5 × 10^6^ sperm/mL at 38.5°C and 5% CO_2_. The five media included: mCCM (Nagashima *et al.* 2015), Fert-TALP (Ferraz *et al.* 2018), mSOF (Hewitt & England 1999), modified NCSU-23 (mNCSU-23 (Petters 1992)), and Tris buffer (Franklin *et al.* 2018) without egg yolk (TRIS) (Supplementary Table 1, see section on supplementary materials given at the end of this article). All media were adjusted to ~300 mOsm (range 295–311 mOsm) and a pH of 7.75–7.8. At 3 h incubation, 6 µg/mL progesterone (or 19 µM, in DMSO, Sigma-Aldrich P8783) was added as a physiological stimulant of acrosome exocytosis (Brewis *et al.* 2001). Incubations were performed in four-well dishes (NUNC, Fisher Scientific). Motility evaluations (% of sperm with a flagellar beating from at least five areas, as previously described (Ferraz *et al.* 2020) were performed on washed sperm at 0, 1, 2, 4, and 18 h. Dishes were gently swirled to mix and detach sperm from the dish, then ~5 µL of the sample was pipetted onto glass slides with a coverslip and evaluated via Brightfield Microscopy (Olympus BX41). The appearance of sperm with hyperactive motility patterns (asymmetrical, rapid flagellar beating (Yanagimachi 1970)) was noted in the treatment groups/time points observed, but could not be accurately quantified due to adherence of sperm heads to the glass slides. At the 0, 1, 2, 4 and 18 h time points, an additional 5 µL of the sample were removed and fixed with 4% paraformaldehyde, and stored at 4°C until evaluation via fluorescence microscopy for acrosome and tyrosine phosphorylation statuses.

In a subset of the animals (*n* = 5), sperm were additionally incubated in either mNCSU-23 or in mNCSU-23 containing only 1 mg/mL BSA without sodium bicarbonate, with osmolarity returned to 300 mOsm with NaCl, to reduce capacitation-stimulation (named non-capacitating mNCSU-23, or NC-mNCSU-23). After 3 h incubation, sperm in each medium were either exposed to 6 µg/mL progesterone (P4) or DMSO vehicle control. Sperm motility, acrosome status, and tyrosine phosphorylation patterns were evaluated as described earlier at hours 1, 2, 4, and 18 of incubation at 38.5°C in 5% CO_2_.

### Fluorescence microscopy

Sperm samples were stained for acrosome (FITC-PNA, ThermoFisher L21409), tyrosine phosphorylation (anti-phosphotyrosine, Millipore Sigma 05-321), and nuclear (Hoechst 33342, Invitrogen H3570) evaluations. Briefly, fixed sperm samples were smeared on glass slides and dried at room temperature. Following three PBS washes, slides were permeabilized with 0.1% Triton-X (Sigma-Aldrich X100) in PBS then blocked in 1% BSA in 0.1% Triton-X PBS for 1 h. Slides were incubated with 2 µg/mL anti-phosphotyrosine antibody in a humidified chamber, either overnight at 4°C or for 4 h at room temperature. Samples were incubated with 1:200 dilution of Alexa rabbit anti-mouse 568, 10 µg/mL FITC-PNA and 5 µg/mL Hoechst 33342 at room temperature for 1 h. After three PBS washes, glass coverslips were mounted with Prolong Anti-fade mountant (ThermoFisher P36980) and slides were observed at 40× magnification with an EVOS Fl Auto 2 (ThermoFisher).

At least 90 sperm were counted for each time point and treatment, per animal. Acrosome status was recorded as intact, partially-intact, or reacted. Localization of phosphorylated tyrosine signal was recorded as in the head, midpiece, tail, tail and midpiece, all, or none of the sperm (Supplementary Fig. 1).

### Statistical analyses

All data were analyzed in R Studio (v. 1.3.1056), with significance set at *P* < 0.05. Motility data (% motile sperm) were evaluated with a nonparametric Wilcoxon test. Tyrosine phosphorylation pattern data were subjected to linear transformation then evaluated via generalized linear mixed effect model with gamma distribution (link log) in package ‘lme4’ (ver. 1.1-23) (Bates *et al.* 2014). Percent reacted acrosome data were logit transformed to achieve normality, then evaluated via linear mixed effect model with r package ‘nlme’ (ver. 3.1-141) (Pinheiro *et al.* 2012). Factors included incubation time, treatment group, the interaction between the treatment group and incubation time, and individual wolf (random factor). For analyses of non-capacitating and capacitating mNCSU-23 data in the presence/absence of P4 and the interaction between treatment group and P4 were also included as fixed effects. *Post hoc* evaluations were done with a nonparametric Wilcoxon test.

## Results

**Figure 1:**
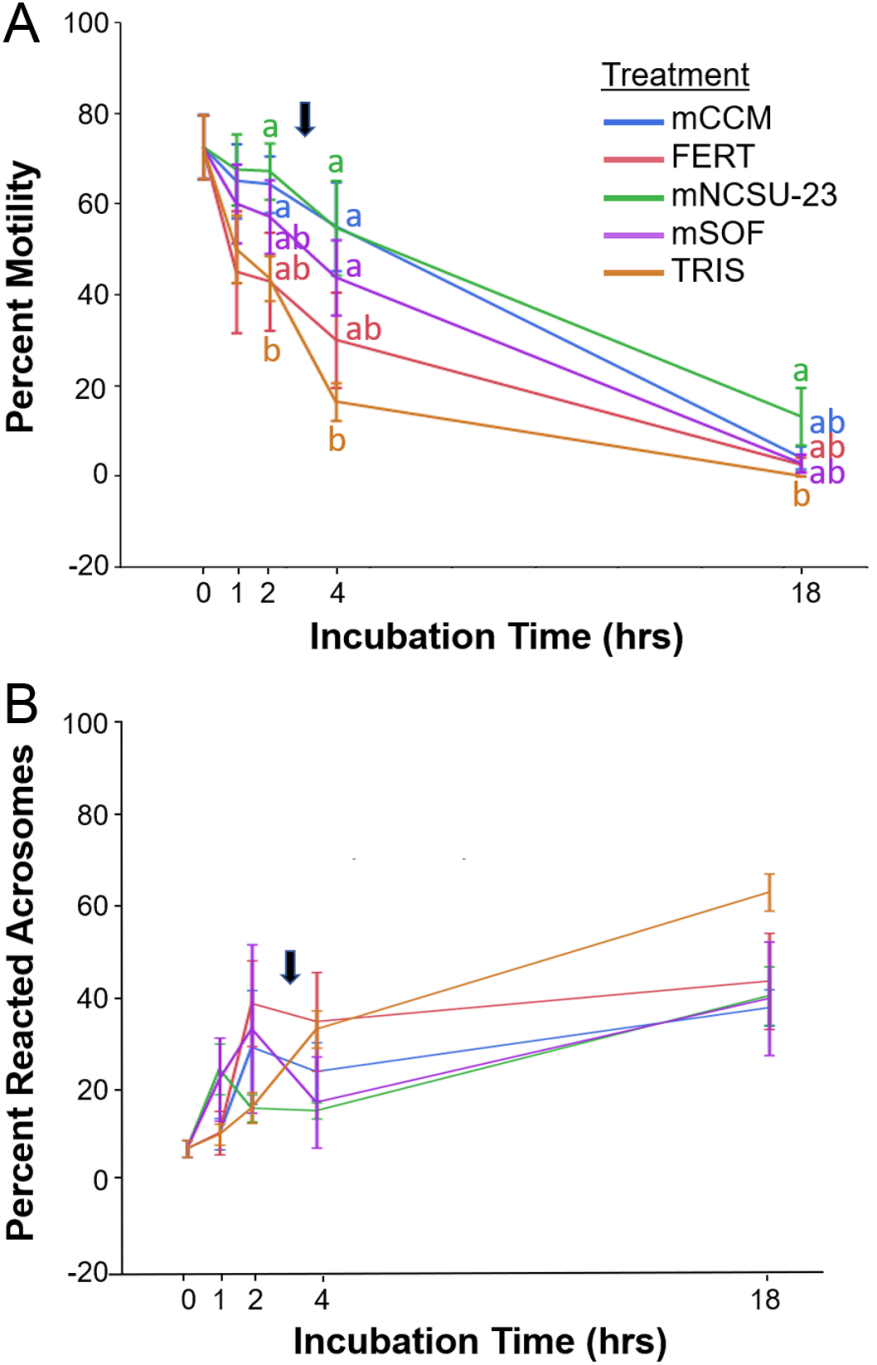
Motility and acrosome status (mean ± s.e.m.) of red wolf sperm in mCCM, FERT, mNCSU-23, mSOF, and TRIS after 1, 2, 4, and 18 h incubation, with (A) percent motile sperm, and (B) percentage of sperm with reacted acrosomal membranes. Letters indicate significant differences between treatments for each time point (*P* < 0.05) and black arrow denotes time of progesterone treatment.

Semen collected via electroejaculation from red wolves ranged from 1.1 to 10.3 mL with a concentration of 18 to 198 × 10^6^ sperm/mL and 30–90% total motile (Table 1). Over the incubation period, sperm motility was better maintained in mCCM and mNCSU-23 compared with FERT and TRIS (Fig. 1A). For example, after 4 h incubation, percent motility (mean ± s.e.) averaged 55.0 ± 9.8 in mCCM and 54.7 ± 10.4 mNCSU-23, respectively, compared with 30 ± 10.5 in FERT and 16.4 ± 4.1% in TRIS. While sperm attachment to glass slides prevented quantification of sperm hyperactive motility, characteristic star-spin movement and lateral head displacement with rapid flagellar beating were observed at the 4 h time point only for mCCM, mNCSU-23 (Supplementary Video 1), and mSOF-treated sperm. By 18 h incubation, mNCSU-23 incubated sperm displayed higher motility than those incubated in TRIS (*P* < 0.05), but was not significantly different from mCCM, mSOF, or FERT incubated sample (*P* > 0.05).

**Table 1:** Summary of semen characteristics following electroejaculation in adult male red wolves.

Collection year	Wolf ID	Age	Volume (µL)	pH	Concentration (sperm/mL)	Total sperm	% motility
2018	2119	3	1056	6.8	18 × 10^6^	19 × 10^6^	75
2018	2118	3	2000	6.8–7.9	28 × 10^6^	56 × 10^6^	70
2018	2075	4	2800	6.8–7.9	198 × 10^6^	554 × 10^6^	80
2019	2208*	2	2000	7.5	25 × 10^6^	50 × 10^6^	30
2019	2206*	2	4310	7	25 × 10^6^	108 × 10^6^	65
2019	2119*	4	2500	NA	34 × 10^6^	85 × 10^6^	90
2019	2118*	4	10,300	7–7.5	59 × 10^6^	608 × 10^6^	90
2019	1790*	9	5000	6–6.5	190.5 × 10^6^	952 × 10^6^	90
Mean			3745.75	7.04	72.2 × 10^6^	304 × 10^6^	73.75
s.e.m.			1040.48	0.15	27.0 × 10^6^	125 × 10^6^	7.12

*Subset of samples which were also assessed under non-capacitating conditions.

**Figure 2:**
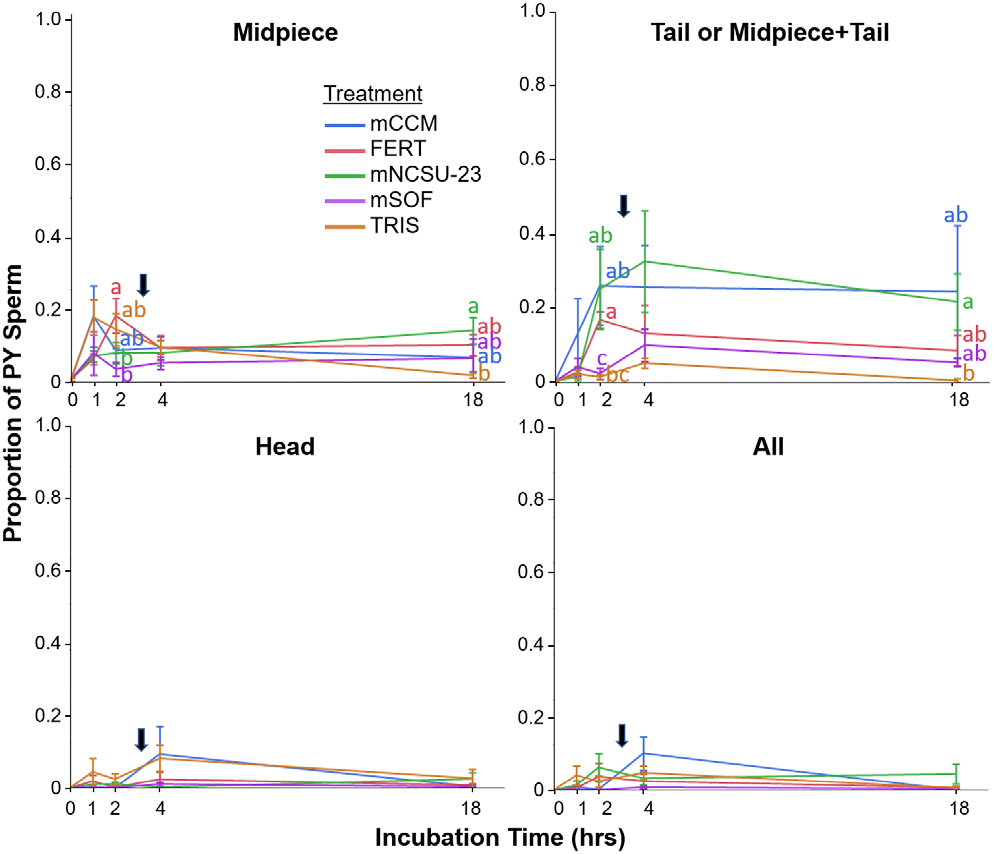
Patterns of tyrosine phosphorylation immunofluorescence (mean ± s.e.m.) in red wolf sperm after 0, 1, 2, 4, and 18 h incubation in mCCM, FERT, mNCSU-23, mSOF, and TRIS medium. Letters indicate significant difference in pattern between treatment groups for each time point (*P* < 0.05) and black arrows denote time of progesterone treatment.

Red wolf sperm acrosomes were evaluated at times 0, 1, 2, 4, and 18 for each wolf and treatment group. Only incubation time (*P* < 0.01), not treatment group, was significantly based on the model. Overall, the proportion of sperm with variable patterns of tyrosine phosphorylation (PY) immunofluorescence increased from 0 to 4 h for all treatments, then reduced from hours 4 to 18. PY signal in the ‘midpiece’ rose from 1 to 2 h of incubation, followed by a more robust increase in ‘tail’ and ‘midpiece + tail’ signal at 2 and 4 h (Fig. 2). At 4 h incubation, there was also a modest but non-significant increase in ‘head’ staining. The most common pattern in the sperm was the tail/midpiece+tail. At 2 h, mCCM, mNCSU, and FERT-incubated sperm displayed significantly higher proportions of sperm with tail/midpiece+tail PY signal compared with mSOF. This was reflective of the overall low proportions of PY signal in sperm incubated in mSOF. For example, at 2 h incubation, between 34 and 40% of sperm in mNCSU-23, mCCM, and FERT displayed PY signal, whereas only ~20% and 6% of TRIS and mSOF, respectively, incubated sperm displayed any PY immunofluorescence.

**Figure 3:**
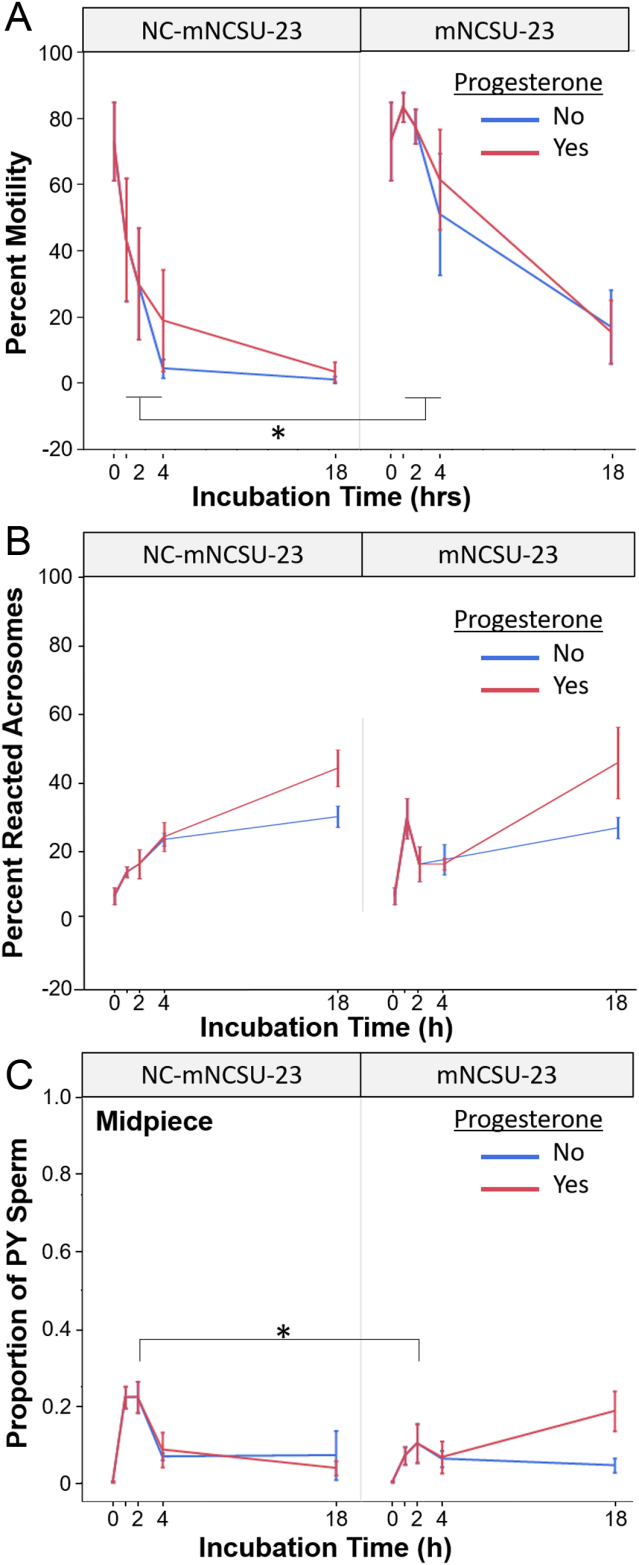
Percent motility (A), acrosome status (B), and proportion of midpiece tyrosine phosphorylation pattern (C) of red wolf sperm incubated under non-capacitating mNCSU-23 (NC-mNCSU-23) or capacitating mNCSU-23 in the absence/presence of progesterone after 3 h *in vitro*.

As mNCSU-incubated sperm consistently displayed high motility, acrosome integrity, and robust PY signaling in extended culture, we selected it for additional comparison of less-stimulating or ‘non-capacitating’ (NC) conditions. A subset of samples (Supplementary Table 2, *n* = 2–5 animals/treatment) were incubated under NC-mNCSU-23 compared with normal, capacitating mNCSU-23. Motility of sperm in NC-mNCSU-23 was significantly reduced compared with mNCSU-23 incubated sperm at hours 1, 2, and 4 of incubation (*P* < 0.05, Fig. 3A). Progesterone supplementation had no influence on percent motility for either media. Sperm in both media displayed a similar loss in acrosome integrity over 18 h culture (*P* < 0.05), with no significant differences between progesterone treatment conditions (Fig. 3B). Incubation time and treatment group were significant in our model for PY patterns (*P* < 0.05), but not progesterone or its interaction with treatment. For example, for midpiece tyrosine phosphorylation, the signal rose in sperm exposed to both treatment conditions at 1–2 h of incubation but was significantly higher in NC-mNCSU-23 incubated sperm compared with mNCSU-23 (*P* < 0.05, Fig. 3C). A similar trend persisted in the other PY patterns (Supplementary Fig. 2), with the overall timeline of signal appearance during incubation similar for NC-mNCSU-23 as the earlier evaluated base medium (Fig. 2).

## Discussion

The ability to maintain red wolf sperm *in vitro* and develop an understanding of capacitation is key to the application of various ART for species conservation. In this study, we sought to (1) identify a base medium supportive of red wolf sperm incubation, and (2) develop a capacitation protocol for red wolf sperm. We found that red wolf sperm motility and acrosome integrity is better maintained in mCCM, mNCSU-23, and mSOF *in vitro* compared with FERT and TRIS solutions. Induction of capacitation, based on patterns of tyrosine phosphorylation signal, occurred between 2 and 4 h *in vitro.* Results indicate that both mNCSU-23 and mCCM are promising sperm handling media, based on their ability to maintain sperm motility and acrosome integrity, and stimulate tyrosine phosphorylation. Our subsequent evaluation of mNCSU-23 indicates additional optimizations are necessary to improve capacitated red wolf sperm’s ability to undergo acrosome exocytosis. Nevertheless, these results represent a key first step toward the recapitulation of capacitation *in vitro,* necessary to produce fertilization-competent sperm for IVF work and to evaluate the quality of post-thaw semen in the future.

In the current study, red wolf sperm characteristics were consistent with what has previously been reported for the population from 1990 to 2004 (Lockyear *et al.* 2016), with motility following electroejaculation ~70% and concentration around 70 million sperm/mL. In much of the previous red wolf semen incubation research thus far, the focus has been on post-thaw motility (Lockyear *et al.* 2009, Franklin *et al.* 2018). Only one study to date has incubated freshly collected red wolf semen, detailing a decrease in motility from ~70 to 55% after 4 h *in vitro* in Tyrode’s albumin lactate pyruvate at room temperature or 0°C (Goodrowe *et al.* 2001). Interestingly, motility of sperm held at 37°C in this study fell to ~40% in the same time frame, and dropped to zero by 10 h *in vitro* incubation, whereas those stored at the lower, non-physiological temperatures maintained ~30% motility for 10 h. As such, we were interested in evaluating different base medium for the maintenance of fresh red wolf sperm function at physiological temperatures. While we still observed a reduction from 74% total motile sperm to 55% after 4 h *in vitro* in mCCM and mNCSU-23 incubated medium at 38°C at 5% CO_2_ in the current study, this is in-line with the better-sustained motility levels observed in ambient or chilled samples previously (Goodrowe *et al.* 2001, Franklin *et al.* 2018). Our results indicate, therefore, that these culture media and/or conditions support the maintenance of red wolf sperm under physiological temperatures *in vitro*.

The appearance of hyperactive motility at 4 h incubation, in combination with elevated midpiece and tail tyrosine phosphorylation patterns, indicates that red wolf sperm capacitation is initiated between 2 and 4 h *in vitro*. This is similar to the finding in domestic dog sperm incubated in mCCM, with the proportion of sperm with hyperactive motility steadily increasing during 1–7 h of incubation (from 20% at 1 h to 80% at 7 h) (Kawakami *et al.* 1998, Kawakami *et al.* 2000). Patterns of tyrosine phosphorylation in the red wolf were also consistent with a previous study in the domestic dog (Petrunkina *et al.* 2003). In the dog, initial PY signal is observed in the midpiece within the first hour of incubation, followed by the signal in the tail between hours 1 and 3*,* and strong signal in the head region primarily after 3 h incubation and a subsequent significant decrease in tail phosphorylation after 6 h (Petrunkina *et al.* 2003). While the same overall pattern was observed in the present study, sperm incubated in mSOF and TRIS displayed less robust tail and midpiece+tail signals. As the TRIS medium was composed only of the base buffer (Franklin *et al.* 2018), with no components added to stimulate capacitation (i.e. BSA or bicarbonate), the lack of signal was not unexpected.

While progesterone itself has been indicated as stimulating capacitation in some species, including the human (de Lamirande *et al.* 1998) and pig (Barboni *et al.* 1995), it did not appear to have an impact on capacitation in the red wolf in our study. Notably, there were no statistically significant differences in motility or tyrosine phosphorylation patterns between P4 and no-P4 treated mNCSU-23 incubated sperm at 4 or 18 h of incubation (Fig. 3 and Supplementary Fig. 2). In non-capacitating mNCSU, the decline in sperm motility/viability in both P4 and no-P4 groups may have been due to poor pH buffering in the absence of bicarbonate, or a need for higher levels of BSA to support red wolf sperm survival *in vitro.* It is possible that, in mNCSU-23 or a more balanced NC-mNCSU-23, the inclusion of additional time-points (i.e. 6 or 8 h) might reveal a delayed effect of progesterone, which should be evaluated in future studies. Alternatively, the red wolf may be similar to the bovine, wherein P4 has been shown to stimulate acrosome reaction but not capacitation in fresh epididymal sperm (Thérien & Manjunath 2003). While progesterone receptors have not been localized on red wolf sperm, work in the domestic dog demonstrated no signal using antibodies against progesterone receptor in un-capacitated sperm, but strong signal over the acrosomal region in capacitated samples (Wu *et al.* 2005). Therefore, we postulate that progesterone is not a key stimulant of sperm capacitation in the red wolf.

Surprisingly, we did not observe an appreciable increase in acrosome-reacted sperm following stimulation with 6 µg/mL (19 µM) progesterone after 3 h incubation (Fig. 1B), despite evidence of capacitation progression. While TRIS- incubated sperm did display an increase in reacted acrosomal membranes from 2 to 4 h incubation, this was mirrored by a significant decline in motile sperm (Fig. 1A) and thereby likely indicative of TRIS-incubated sperm becoming non-viable. Based on domestic dog work (Sirivaidyapong *et al.* 1999, Brewis *et al.* 2001), we anticipated significant induction of acrosomal exocytosis in progesterone-treated sperm within 20 min to 1 h of incubation, assuming they had achieved capacitation. Thus, in the subset of samples incubated under ‘non-capacitating’ mNCSU-23 and mNCSU-23, we expected a robust acrosome reaction to progesterone in the latter compared with the former medium at 4 h incubation. Instead, we observed similar acrosome patterns for both media with or without progesterone, suggesting that either red wolf sperm capacitation was not sufficiently achieved after 3 h incubation and/or the use of progesterone for stimulation of acrosomal exocytosis was not optimal for this species. The ~19 µM concentration of progesterone utilized here was slightly higher than what has previously been utilized for domestic dog sperm, ranging typically from 10 µM (Sirivaidyapong *et al.* 1999, Deppe *et al.* 2010) to 13 µM (Brewis *et al.* 2001); however, it is in range with dog follicular fluid, which is ~2.6 µg/mL (~8 µM) before the luteinizing hormone (LH) surge, rising to ~11.7 µg/mL (~37 µM) post-LH surge (Fahiminiya *et al.* 2010). As the progesterone concentration in the oviductal fluid around the time of fertilization in canid species is not yet known, it is possible that the concentrations applied here were not optimal for induction of acrosome exocytosis. Alternatively, a previous study in the silver fox (*Vulpes vulpes*) has reported that sperm require 5–8 h to capacitate and subsequently successfully penetrate fox ova (Feng *et al.* 1994). Moving forward, studies should determine if red wolf sperm require longer incubation for *in vitro* capacitation prior to stimulation to induce acrosome exocytosis, more frequent evaluation following stimulation to assess effects, or if alternative/additional stimulants (i.e. zona pellucida proteins (Brewis *et al.* 2001)) are more physiologically relevant than progesterone for induction of the acrosome reaction in this species.

Similarly, the investigation into the effects of specific medium components may be warranted to further optimize the capacitation of red wolf sperm. For example, both mNCSU and mCCM contained relatively high levels of glucose (5.55 and 2.78 mM, respectively), compared with mSOF and FERT (1.50 and 0.0 mM, respectively – see Supplementary Table 1). Glucose supplementation has been demonstrated to extend the survivability of human sperm (Amaral *et al.* 2011) and induce hyperactivation-like changes in motility in domestic dog sperm (Rigau *et al.* 2001). We postulate that these higher glucose concentrations in mNCSU and mCCM may have had a similar supportive effect in the red wolf sperm. This effect was likely absent in TRIS-incubated sperm owing to the lack of other supportive components, such as BSA. Similarly, the absence of additional amino acids in mCCM compared with mNCSU did not appear to significantly influence any of the sperm metrics evaluated in this study. Though taurine and hypotaurine have been demonstrated to improve mammalian sperm motility *in vitro* (Meizel *et al.* 1980, Alvarez & Storey 1983), the lack of significant differences between mCCM and mNCSU-incubated sperm suggests these factors may not be as influential in the red wolf. Still, with the identification of a supportive base medium here, we can now adjust these specific components to better understand the mechanisms and improve red wolf sperm capacitation.

To date, few studies have specifically assessed sperm capacitation in non-domestic canid species. The paucity of work in this field is due in part to the difficulty in obtaining fresh semen samples to study, particularly in endangered canid species. Nevertheless, with 11 of the 36 extant species of canid listed as either endangered or near threatened (IUCN 2020), the development of genome preservation and assisted reproductive technologies for canids is becoming increasingly important to species survival (Luvoni *et al.* 2006). In this study, we have identified two base media (mCCM and mNCSU-23) capable of supporting *in vitro* incubation of red wolf sperm at physiological temperatures. Further, we have described patterns of tyrosine phosphorylation during the early stages of capacitation in the red wolf. While further studies are necessary to develop *in vitro* capacitation for application to IVF or for the evaluation of sperm fertilizing capacity post-thaw, our findings represent an important step in the development of assisted reproductive technologies in the critically endangered red wolf.

## Supplementary Material

Supplemental Figure 1. Example images of red wolf sperm fluorescence microscopy, with intact, partially reacted, and reacted acrosomal membranes (green), and tyrosine phosphorylation (red) patterns of head, midpiece, tail, all (head + midpiece + tail), and none, and DAPI (blue) for nuclear content.

Supplemental Figure 2. Patterns of tyrosine phosphorylation immunofluorescence (mean ± SEM) in red wolf sperm after 0, 1, 2, 4, and 18 hrs incubation in NC-mNCSU-23 and mNCSU-23 medium. Asterisk (*) indicates significant differences between medium treatment groups at a given time point (P < 0.05).

Supplemental Table 1. Formulations for media used for red wolf sperm incubation

Supplemental Table 2. Sample sizes of red wolf sperm incubated in NC-mNCSU-23 and mNCSU-23 in the absence/presence of progesterone supplementation after 3 hrs.

Supplemental Video. Example video of hyperactivated motility patterns, from red wolf #2118 sperm incubated for 4 hrs in mNCSU-23.

## Declaration of interest

The authors declare that there is no conflict of interest that could be perceived as prejudicing the impartiality of the research reported.

## Funding

This work was supported by the Volgenau Foundation, Brandt Foundation, Point Defiance Zoo and Aquarium’s Dr Holly Reed Conservation Fund, the Friends of the National Zoo (FONZ), and the Smithsonian Institution http://dx.doi.org/10.13039/100000014.

## Author contribution statement

J B N, M d A M M F and N S conceived the study. J B N, M d A M M F, and S H K performed the experiments. J B N analyzed the data. J B N, M d A M M F, S H K, and N S wrote the paper.
