# Supplementary figures and images for: Investigating media that support red wolf (Canis rufus) sperm viability and capacitation in vitro
Source: Reprod Fertil. 2020 Dec 28;1(1):83–92. doi: 10.1530/RAF-20-0042 (PMC8812450; doi:10.1530/RAF-20-0042)

Acrosome

Tyr  
Phosphorylation

Merged Examples

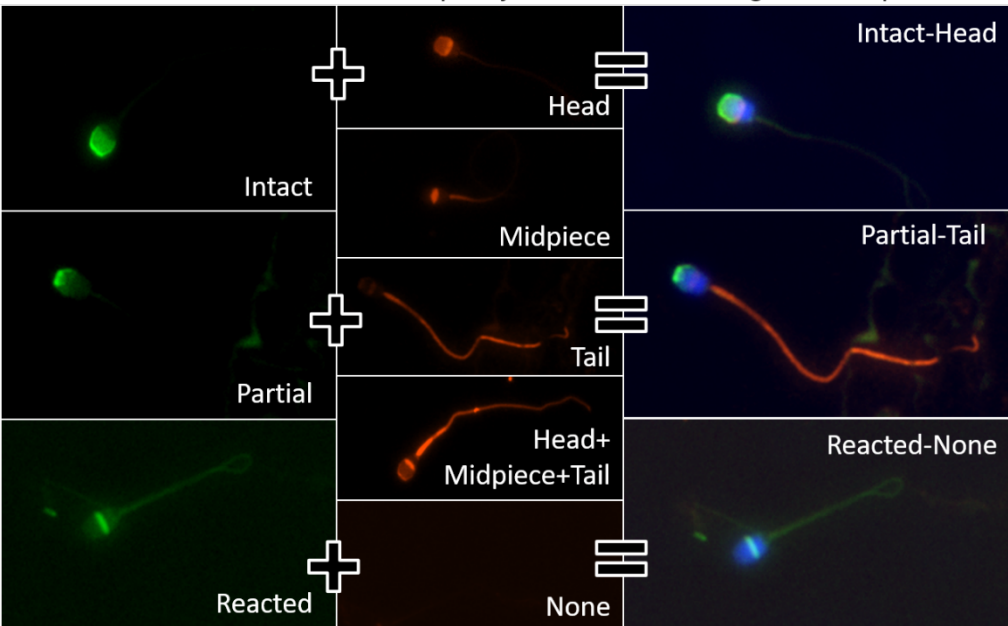

Supplement: Supplemental Figure 1. Example images of red wolf sperm fluorescence microscopy, with intact, partially reacted, and reacted acrosomal membranes (green), and tyrosine phosphorylation (red) patterns of head, midpiece, tail, all (head + midpiece + tail), and none, and DAPI (blue) for nuclear content. [file supplementary_figure_1.pdf]

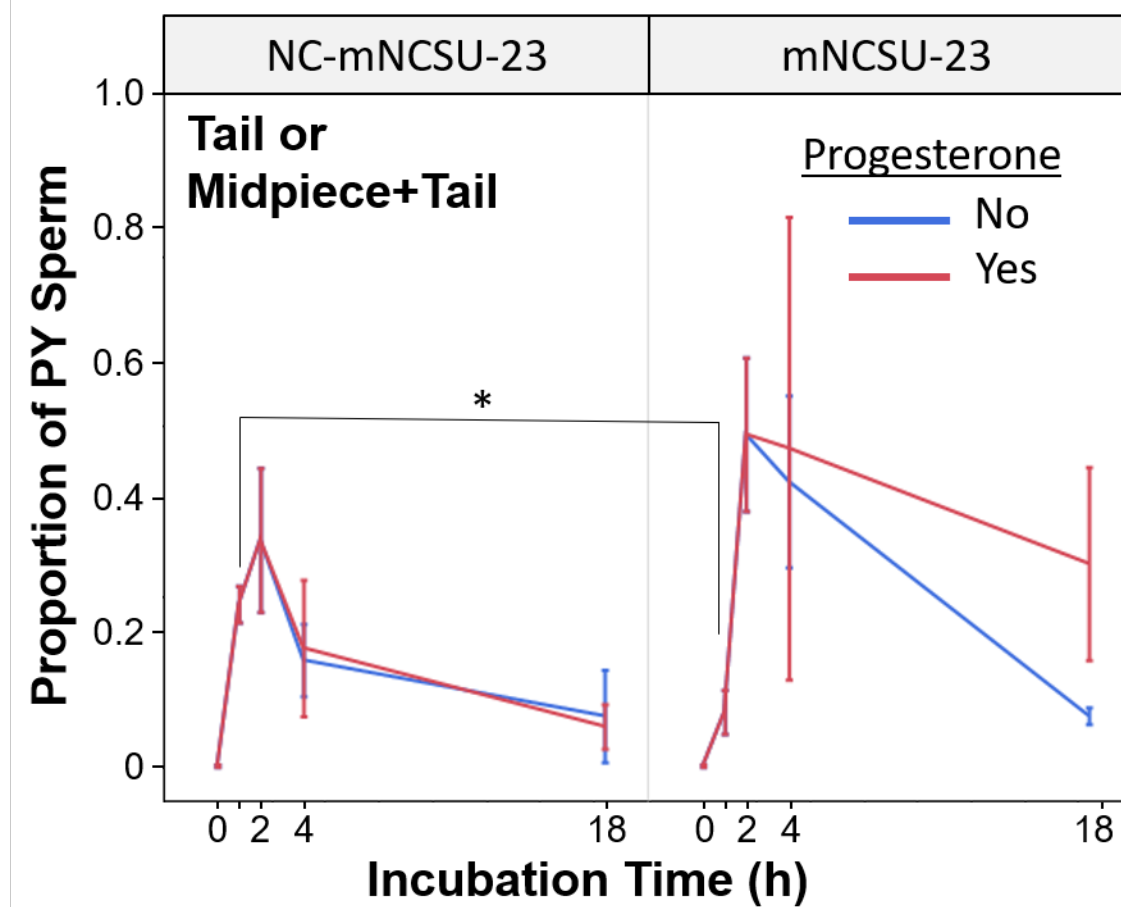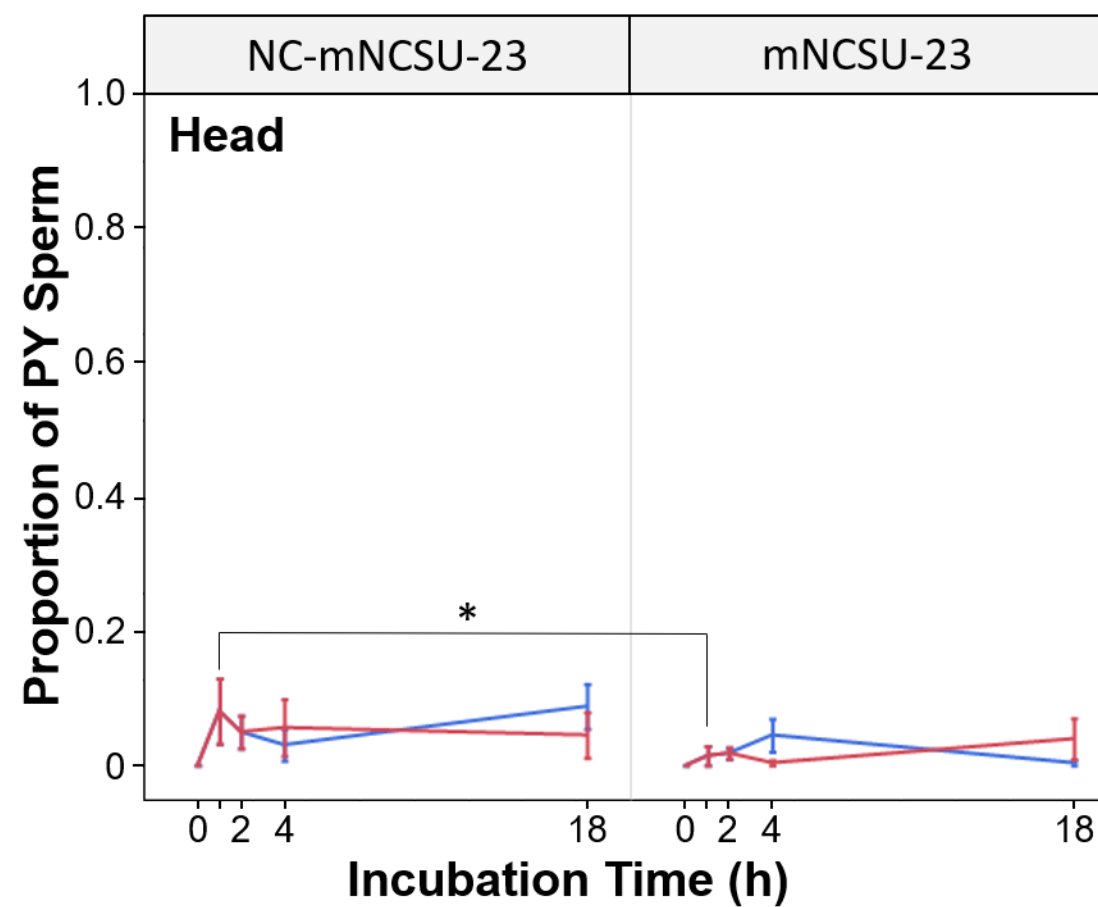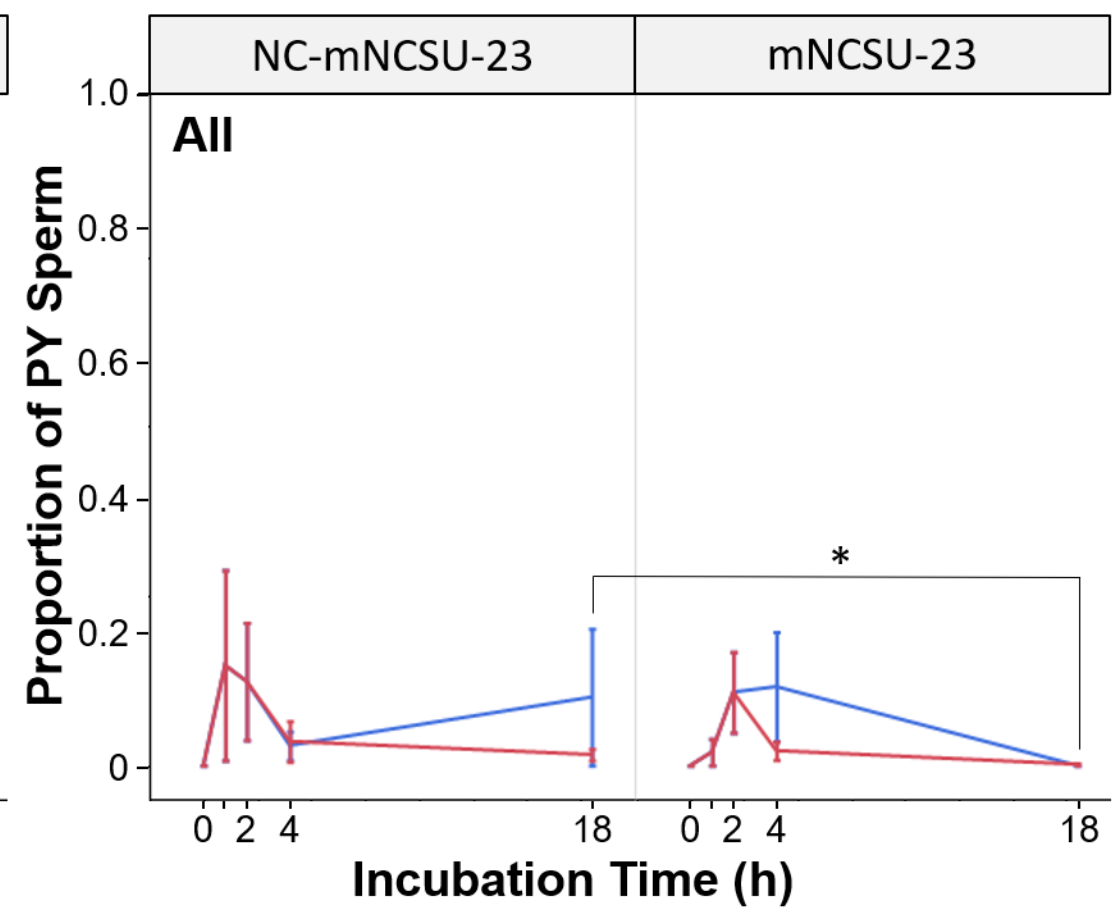

Supplement: Supplemental Figure 2. Patterns of tyrosine phosphorylation immunofluorescence (mean ± SEM) in red wolf sperm after 0, 1, 2, 4, and 18 hrs incubation in NC-mNCSU-23 and mNCSU-23 medium. Asterisk (*) indicates significant differences between medium treatment groups at a given time point (P < 0.05). [file supplementary_figure_2.pdf]
