# Supplementary material for: Investigating media that support red wolf (Canis rufus) sperm viability and capacitation in vitro
Source: Reprod Fertil. 2020 Dec 28;1(1):83–92. doi: 10.1530/RAF-20-0042 (PMC8812450; doi:10.1530/RAF-20-0042)
Supplement: Supplemental Table 1. Formulations for media used for red wolf sperm incubation [file supplementary_table_1.pdf]

Supplemental Table 1. Formulations for media used for red wolf sperm incubation

	**Concentration (mM)**				
**Reagent**	**mCCM**	**FERT**	**mNCSU-23**	**mSOF**	**TRIS**
NaCl	83.49	114.00	108.73	107.70	-
KCl	4.78	3.20	4.78	7.16	-
$CaCl_2$	1.71	2.00	1.70	1.71	-
KH_2PO_4	1.19	-	1.19	1.19	-
$MgCl_2$	1.00	0.50	1.19	0.49	-
Glucose	2.78	-	5.55	1.50	55.00
Na pyruvate	0.25	0.20	0.02	0.33	-
Taurine	-	-	7.00	-	-
Hypotaurine	-	0.01	5.00	-	-
Glutamine	-	-	1.00	-	-
Na bicarbonate	37.61	25.00	25.07	25.07	-
BSA	2 mg/ml	6 mg/ml	4 mg/ml	32 mg/ml	-
Penicillin / Strep (100X)	100 U/ml	100 U/ml	100 U/ml	100 U/ml	100 U/ml
HEPES	25.00	-	-	-	-
Lactate	21.55	11.00	-	3.30	-
Heparin	-	10 µg/ml	-	-	-
D-penicillamine	-	0.02	-	-	
Epinephrine	-	0.001	-	-	-
NaH_2PO_4	-	0.40	-	-	-
TRIS	-	-	-	-	220.00
Citric acid	-	-	-	-	66.00
