# Supplementary material for: Investigating media that support red wolf (Canis rufus) sperm viability and capacitation in vitro
Source: Reprod Fertil. 2020 Dec 28;1(1):83–92. doi: 10.1530/RAF-20-0042 (PMC8812450; doi:10.1530/RAF-20-0042)
Supplement: Supplemental Table 2. Sample sizes of red wolf sperm incubated in NC-mNCSU-23 and mNCSU-23 in the absence/presence of progesterone supplementation after 3 hrs. [file supplementary_table_2.pdf]

Supplemental Table 2. Sample sizes of red wolf sperm incubated in NC-mNCSU-23 and mNCSU-23 in the absence/presence of progesterone supplementation after 3 hrs.

Incubation Time (hrs)	P4 (yes/no)	NC-mNCSU-23	mNCSU-23
0	-	5	5
1	-	4	4
2	-	4	4
4	No	5	4
4	Yes	4	2
18	No	2	2
18	Yes	3	4
